# Health Tract, No. 273

**Published:** 1867-02

**Authors:** 


Vol. XIV. FEBRUARY, 1867. No. 2.

HEALTH TRACT, No. 273.

### ILLOGICAL SEQUENCES.

"A boy died lately in Chicago from the effects of swimming in a pond where the carcasses of animals had been deposited."

Such is the assertion of a scientific publication. A simple statement of facts, without inductions, or inferences, or mere opinion is an invaluable faculty possessed by perhaps one in a thousand. Multitudes of fatal errors in relation to health and life are thrown upon the world from time to time by thoughtless or ignorant writers. Only men of thought, of high mental discipline are really capable of a safe use of facts; it requires a logical mind and great powers of close and accurate observation.

The reader may owe his life to the proper interpretation and practical use of the idea of this article.

The best way of stating the fact above would have been: "A boy died after swimming in a pond where animal carcasses had been deposited."—That is the bald statement. No one knows from this single instance whether he died from staying in too long, or from over-exertion in the water; or from remaining in until he was chilled; or from having gone in while heated; or from going in after a hearty meal. Death has frequently followed from each one of these causes. There are five chances to one that death did not result from decaying carcasses in the water. The daily papers announced yesterday that a woman "caught the cholera" from handling the clothing in which a cholera patient had died. Here is a fact and a theory so intimately conjoined that thousands of indiscriminating minds and other thousands who are too inert to investigate, would lay down the paper and adopt as a life-long sentiment, that "cholera was catching" from handling the clothing of the diseased, while the whole medical world are divided point-blank on that subject. The facts of the case were, an old woman was made drunk and persuaded by the wife of the deceased to go by night and dig up the clothing which had been buried by order of the Board of Health, to prevent a supposed cause of spreading the disease. The age of the woman, the fatigue, the drunkenness, the unusual exposure or exercise, were each a possible cause of death.

It was lately stated that a mad-stone* had been applied to a mad dog's bite in eighteen cases, and not one had been attacked with hydrophobia. The conclusion of nine minds out of ten would be that the mad-stone cured hydrophobia, but neither case was attacked, and the greatest surgeon of his age, John Hunter, stated that out of twenty-one persons bitten by a dog known to be mad, only one became hydrophobic; and it is not stated that anything was done for either of them. If to a man suffering from actual hydrophobia, the mad-stone was applied and nothing else was done, and he got perfectly well, that would be a practical fact of great value. We have never seen any such case recorded. Persons may lose life by losing time in the employment of an inefficient remedy, to the neglect of those of known value.

*The Mad-Stone is said to be obtained from the part of the deer called the rennet, and is larger in the older animals.

